# Genome-Wide Identification and Characterization of Histone Acetyltransferases and Deacetylases in Cucumber, and Their Implication in Developmental Processes

**DOI:** 10.3390/genes16020127

**Published:** 2025-01-23

**Authors:** Agnieszka Skarzyńska-Łyżwa, Szymon Turek, Maksymilian Pisz, Aparna, Wojciech Pląder, Magdalena Pawełkowicz

**Affiliations:** Department of Plant Genetics, Breeding and Biotechnology, Institute of Biology, Warsaw University of Life Sciences-SGGW, 159 Nowoursynowska Str., 02-776 Warsaw, Poland

**Keywords:** epigenetics, histone acetyltransferase, histone deacetylase, chromatin modification, plant development, cucumber

## Abstract

Background/Objectives: Cucumber (*Cucumis sativus*) provides a model for exploring the molecular basis of sex determination, particularly the regulation of floral organ differentiation through gene expression. This complex process is modulated by epigenetic factors, such as histone acetyltransferases (HATs) and histone deacetylases (HDACs), which respectively activate and repress gene transcription by adding or removing acetyl groups from histone proteins. Despite their known functions, the roles of HATs and HDACs throughout cucumber’s floral developmental stages remain unclear. Methods: In this study, we conducted a genome-wide analysis of *HAT* and *HDAC* gene families in cucumber, examining their phylogenetic relationships, gene structures, protein domains, and expression profiles across various stages of floral development. Results: We identified 36 *CsHAT* and 12 *CsHDAC* genes, grouping them into families with evolutionary counterparts in other plant species. RNA sequencing revealed stage-specific expression patterns, suggesting dynamic roles for these gene families in floral organ development. Conclusions: These findings contribute valuable insights into the epigenetic regulation of gene expression in cucumber flower formation, presenting avenues for further research on the genetic control of plant reproductive development.

## 1. Introduction

Chromatin, a highly intricate and organized structure, is composed of densely packed DNA molecules within eukaryotic nuclei. The fundamental unit of chromatin is the nucleosome, which consists of approximately 146 base pairs of DNA intricately wound around an octamer of histone proteins [1]. This octamer comprises four distinct histone subunits, namely H2A, H2B, H3, and H4. Post-translational modifications (PTMs) occur on specific amino acid residues located on the unstructured tails of these histone proteins [2]. These PTMs, such as acetylation, methylation, phosphorylation, ubiquitination, ADP-ribosylation, and glycosylation, play pivotal roles in epigenetic regulation of gene expression [3,4].

Among these modifications, acetylation stands out as one of the extensively researched mechanisms. Notably, lysine residues in the histone proteins H3 and H4 are frequently targeted for acetylation [5,6]. It is important to note that epigenetic modifications do not involve changes in the underlying DNA sequence itself. Instead, they primarily involve modifications of histone proteins and DNA methylation. These epigenetic modifications exert profound influences on gene expression and are instrumental in facilitating adaptive responses in plants, enabling them to thrive in diverse developmental phases and under challenging environmental conditions [7,8].

The acetylation process involves combining two different counteracting enzymes, histone acetyltransferases (HATs) and histone deacetylases (HDACs). Both enzymes work in a reversible manner [9]. HATs, through their enzymatic action, facilitate the loosening of the interactions between DNA and histones. This enzymatic activity results in the relaxation of chromatin structure, rendering it more accessible for gene expression [10]. This chromatin relaxation phenomenon is instrumental in promoting gene transcription, as it frees the DNA from its tightly wound configuration, allowing transcriptional machinery to bind more effectively. Conversely, HDACs catalyze the removal of acetyl groups from histone proteins. This deacetylation process leads to the condensation of the chromatin structure, making it more compact. In addition to promoting chromatin condensation, HDACs also exert an inhibitory effect on gene transcription [11].

Both HATs and HDACs can be categorized into distinct families. In plants, HATs contain four families such as HAG of the GNAT (GCN5-related N-terminal acetyltransferases) superfamily, HAC of the p300/CBP (CREB-binding protein) family, HAM of the MYST (Moz, ybf2/saa2 and Tip60) superfamily, and HAF of the TAFII 250 (TATA-binding protein-associated factors) family, while HDACs are divided into three families such as RPD3/HDA1 (Reduced Potassium Dependency 3/Histone Deacetylase 1), SIR2 (Silent Information Regulator 2), and HD2 (Histone Deacetylase 2) [12,13].

HATs and HDACs perform crucial roles in the developmental processes of plants, including flowering timings and regulation of seed, flower, leaf, and root growth, as well as fruit development and ripening-related phytohormone metabolism and plant responses to biotic and abiotic environmental stresses [14,15].

This study aimed to identify and subsequently analyze histone modifiers (CsHATs and CsHDACs) found in the genome of the B10 line of cucumber. A comprehensive analysis of the structure, phylogeny, and composition of this family is presented in this research. We also identified the expression levels of different *HATs* genes (*CsHATs)* and *HDACs* genes *(CsHDACs)* at various developmental stages of the studied cucumis B10 line. 

## 2. Materials and Methods

### 2.1. Identification of Histone Acetyltransferases and Deacetylase Protein Sequences

Protein sequences of cucumber acetyltransferases and histone deacetylases were identified by searching the B10v3 genome annotation, as established and published by Osipowski and coauthors [16].

Additionally, a BLASTP [17] analysis was performed on the B10 proteins against related cucumber proteomes, in particular the 9930 v3 line [18] and the Gy14 line [19]. These searches yielded a consolidated list of shared proteins across the three cucumber lines, focusing on those annotated as histone acetyltransferases and deacetylases [12,20]. The InterProScan program [21] was used to analyze the protein sequences of the B10v3. All databases available in the local version of the program were utilized, consisting of TIGRFAM, SFLD, SUPERFAMILY, PANTHER, Gene3D, Hamap, ProSiteProfile, Coils, SMART, CDD, PRINTS, PIRSR, ProSitePattern, AntiFam, Pfam, MobiDBLite, and PIRSF. This comprehensive search expanded the HATs list by identifying additional proteins associated with characteristic domains or families, such as GNAT, MYST, and CBP.

Subsequently, the results from InterProScan were used to refine the classification. Proteins were narrowed down to those containing the specific domains relevant to this category, ensuring a more targeted and accurate dataset.

Using the identified protein domains and the family classifications obtained from the Pfam and Superfamily programs, HAT proteins were categorized into the HAG, HAC, HAF, and HAM groups. This approach enabled the classification of cucumber HDACs into three distinct groups: HDA, SRT, and HDT, reflecting their sequence similarity and domain characteristics.

### 2.2. Multiple Sequence Alignment and Phylogenetic Analysis

The amino acid sequences of the *HAT* and *HDAC* genes were aligned using Clustal W 2.1 [22]. The neighbor-joining method was employed with 1000 replications of bootstrapping and partial deletion. Selected histone acetyltransferase and deacetylase sequences from cucumber, *Arabidopsis thaliana*, and *Solanum lycopersicum* were used to perform the MSA. In the next step, the IQ-TREE server [23] was used to construct a phylogenetic tree based on the sequence alignment file. Finally, the iTOL server [24] was used to visualize the resulting tree and present it in a suitable format. This process was then repeated to compare previously identified domain sequences in the same way.

### 2.3. Protein Motif Analysis and Determination of Physicochemical Properties of CsHATs and CsHDACs 

The conserved motifs identification was performed with MEME [25]. For CsHATs, the number of searched motifs was set to 30, while for CsHDACs, this value was set to 20. The results of the motifs search were visualized with TBtools. The amino acid length, molecular weight (Mw), and isoelectric point (pI) of the identified CsHATs and CsHDACs proteins were estimated using the EMBOSS PEPSTATS tool [26]. Additionally, the WoLF PSORT online tool [27] was used to predict the sub-cellular localization of the CsHATs and CsHDACs proteins.

### 2.4. Chromosomal Localization and Gene Structure

Chromosomal localization was performed based on the B10v3 cucumber genome. Visualization was performed with the circlize [28] package for R with a custom script. Gene structure information was obtained from B10v3 genome annotation [16,29] and visualized using TBtools [30].

### 2.5. Expression Analysis of CsHATs and CsHDACs 

The RNA-seq data analyzed in this study was sourced from the SRA database under the identifier PRJNA1166086. Reads were aligned to the B10v3 cucumber genome sequence (GenBank: LKUO00000000.3). Gene expression levels were quantified using the Salmon software [31], with sequence-specific and GC content bias correction. Normalization was performed to Transcripts Per Million (TPM), and differential expression analysis was conducted using the Limma package [32]. Genes identified as acetyltransferases and deacetylases were extracted from the TPM matrix, and a heatmap of their expression profiles was generated with the gplots package [33].

Differential gene expression results were filtered for genes of interest (acetyltransferases and deacetylases) using an adjusted *p*-value threshold of 0.05. Venn diagrams were employed to visualize differentially expressed genes (DEGs) across floral bud growth stages, including comparisons such as 1–2 mm vs. shoot apex, 3–5 mm vs. 1–2 mm, 6–8 mm vs. 3–5 mm, 9 mm vs. 6–8 mm, and comparison of each developmental stage relative to the leaf stage.

### 2.6. Cis-Acting Elements in Promotor Regions

To identify the *cis*-acting elements in the promoter region of *CsHATs* and *CsHDACs* genes, the upstream sequences of 1000 bp were extracted. The sequences were analyzed using the PlantCARE database [34] to predict *cis*-regulatory elements. The results were then organized and visualized using TBtools.

## 3. Results

### 3.1. Identification of CsHATs and CsHDACs in C. sativus

Analysis of the B10v3 cucumber database led to the identification of 36 CsHATs and 12 CsHDACs. Among the CsHATs, 28 of the proteins were classified as belonging to the HAG family, seven of them were assigned to the HAC family, and a single protein was assigned to the HAM family. None of the identified CsHAT proteins was found to be a part of the HAF family. For the CsHDACs, nine proteins were assigned to the HDA family, three proteins were identified in the HDT family, and no protein was found in the SRT family. Additionally, one CsHAT and one CsHDAC were found only in the 9930 line. The obtained results were compared with existing protein distribution data for *A. thaliana* and *S. lycopersicum* and compiled in Table 1.

### 3.2. Comparative Phylogenetic Analysis

**Figure 1:**
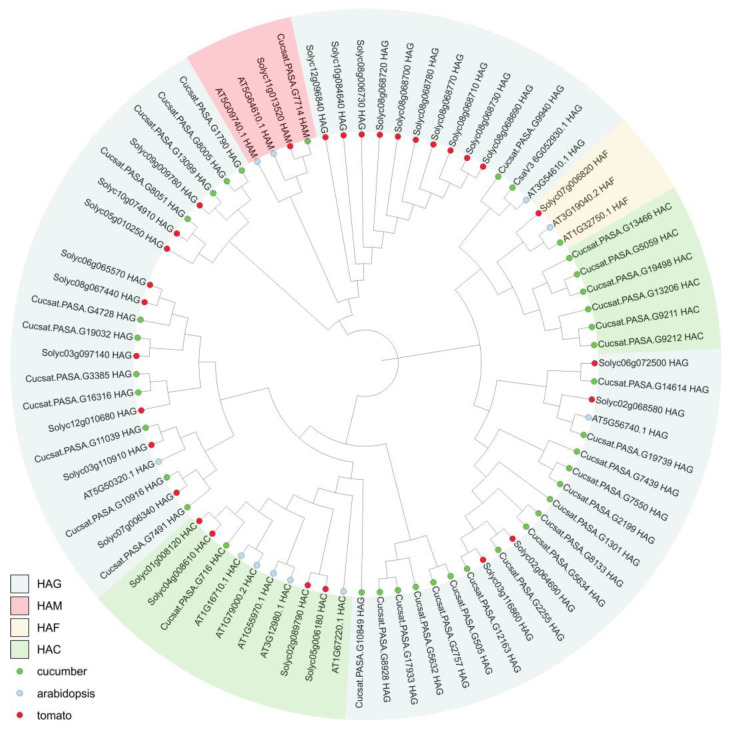
Phylogenetic analysis of HAT proteins from *Cucumis sativus* (36 proteins, indicated by green circles), *Arabidopsis thaliana* (12 proteins, indicated by blue circles), and Solanum lycopersicum (29 proteins, indicated by red circles). The phylogenetic tree was built using the neighbor-joining method. The HAG, HAM, HAF, and HAC families have been marked with different colors.

We compared the 36 CsHAT proteins from cucumber that were identified in this study with 12 proteins previously identified in *A. thaliana* [12] and 29 proteins identified in the *S. lycopersicum* [20]. To investigate the protein relationships, all described HAT proteins were used to construct the phylogenetic tree based on the neighbor-joining method (Figure 1). Proteins that were classified as belonging to the same family (HAG, HAM, HAC, or HAF) were mostly grouped together on the same branch of the tree.

**Figure 2:**
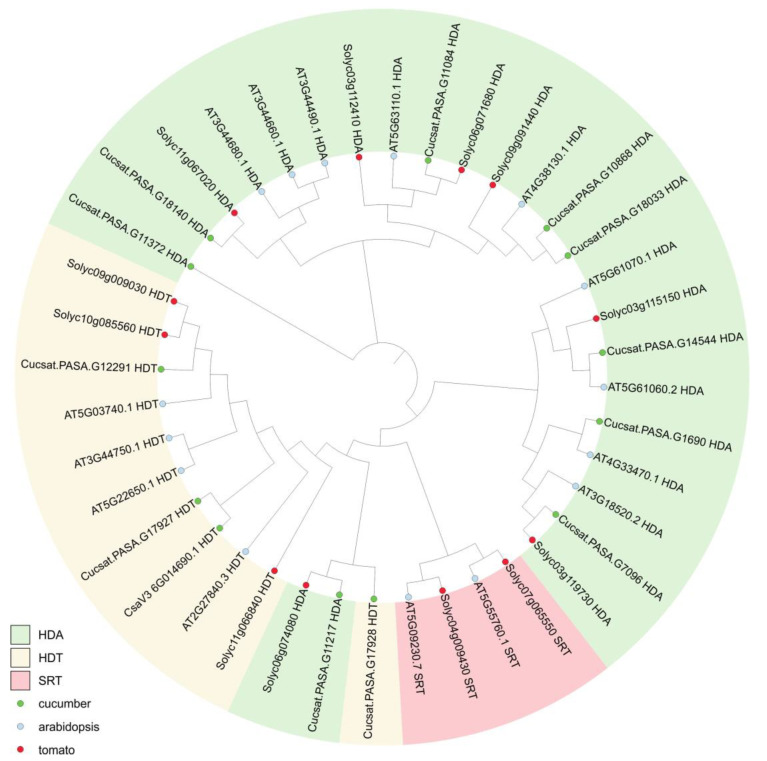
Phylogenetic analysis of HDAC proteins from *Cucumis sativus* (12 proteins, indicated by green circles), Arabidopsis thaliana (15 proteins, marked with blue circles), and Solanum lycopersicum (12 proteins, marked with red circles). The phylogenetic tree was built using the neighbor-joining method. The HDA, HDT, and SRT families were indicated by different colors.

We performed the same analysis for HDACs proteins—12 proteins identified in the cucumber, 15 proteins from *A. thaliana,* and 12 proteins from *S. lycopersicum*. All the sequences were used to prepare a neighbor-joining phylogenetic tree (Figure 2). Proteins assigned to the same family (HDA, HDT, or SRT) were grouped together on the same branch, except for two HDA proteins (one from tomato and one from cucumber) that were grouped together with the HDT family.

Based on the InterProScan protein domain identification (Supplementary Table S1), we constructed the phylogenetic trees using only domain sequences specific to each protein. We used the neighbor-joining method. We used 77 sequences for HATs and 39 sequences for HDACs. The tree obtained for the HAT proteins showed similar clustering to the alignment prepared using whole protein sequences. The tree obtained for HDAC proteins also showed similar clustering. However, the domain alignment allowed for better grouping of the HDT family proteins, which were distributed on the same branch except for one protein from the cucumber. The resulting phylogenetic trees based on the specific domain sequences are presented in Figure S1 in the Supplementary Materials Section.

### 3.3. Characteristics of Motifs, Domains, Gene Structure of CsHATs and CsHDACs

**Figure 3:**
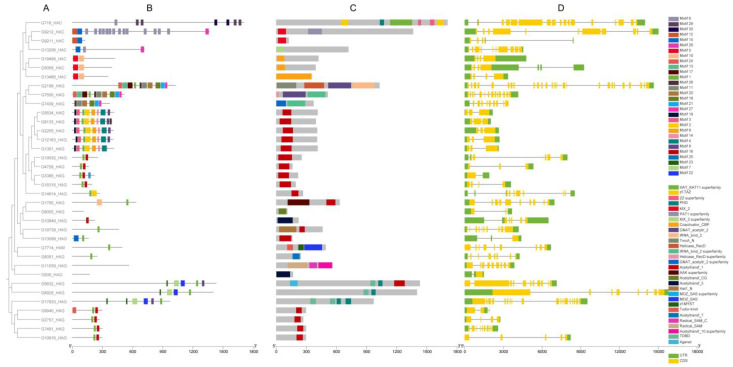
Phylogenetic relationship, conserved motifs, protein domain distribution, and gene structure of identified *CsHAT* genes. (**A**) The phylogenetic tree was performed with the N-J method. (**B**) Motif composition predicted using MEME. The 30 identified motifs are indicated by boxes. (**C**) Protein domain distributions were constructed based on the pfam database. The 30 domains are indicated by colored boxes. (**D**) CsHAT gene structures were constructed based on the B10v3 genome annotation. Yellow boxes indicate exons, black lines indicate introns, and green boxes indicate UTRs.

For a better understanding of the structural diversity of the CsHAT and CsHDAC families, we identified and analyzed the conserved motifs using the MEME tool and analyzed the occurrence of conserved protein domains with InterProScan. We identified 30 conserved motifs in the CsHAT proteins, ranging from 7 aa to 50 aa in length, and 30 protein domains from the Pfam database (Figure 3). There is no clear pattern for each HAT class in terms of the predicted motifs. However, the proteins that clustered together on the same branches of the phylogenetic tree showed a similar motif distribution. For example, HAC family proteins G19498, G5059, and G13466 consist only of motif 5 and motif 10, which are related to the occurrence of the KIX domain, a coactivator of CBP, found only in these three proteins. Another group is the HAG family proteins G5634, G8133, G2255, G12163, and G1301, which showed a similar composition of conserved motifs, which are motifs 1–4, 8–9, 14, and 19. Motif 1 was found in 24 CsHAT proteins. This motif is most likely related to the occurrence of the acetyltransferase 1 domain.

**Figure 4:**
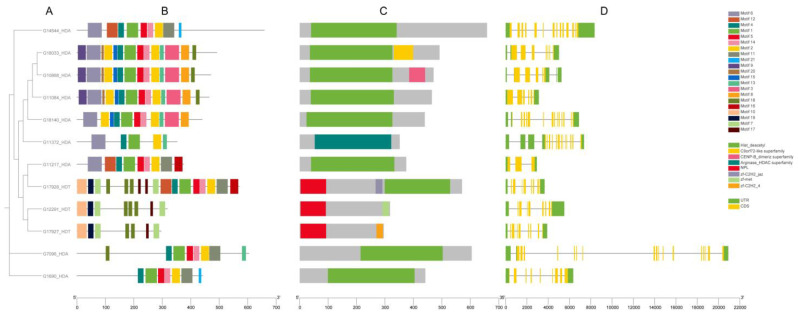
Phylogenetic relationship, conserved motifs, protein domain distribution, and gene structure of identified *CsHDAC* genes. (**A**) The phylogenetic tree was made with the N-J method. (**B**) Motif composition predicted using MEME. The 21 identified motifs are indicated by boxes. (**C**) Protein domain distribution was created using the pfam database. Eight domains are marked with colored boxes. (**D**) CsHDAC gene structures were constructed based on the B10v3 genome annotation. Yellow boxes indicate exons, black lines indicate introns, and green boxes indicate UTRs.

Through analysis of the conserved motifs in the CsHDAC proteins, we identified 21 motifs ranging from 9 aa to 50 aa in length and seven domains from the Pfam database (Figure 4). Motifs 7, 10, and 19 are specific to the HDT protein family. They are associated with the presence of nucleoplasmin-like domain (NPL). The HDA proteins showed a similar composition of conserved motifs. Each of them has motifs 1, 2, 4, 5, and 14 (with the exception of protein G11372, which lacked motifs 5 and 14), which are most likely related to the histone deacetylase domain found in almost all protein sequences analyzed.

The gene structure analysis was conducted based on the cucumber B10v3 genome annotation (Supplementary Table S2). The *CsHAT* genes varied in length (the shortest transcript consisted of 406 bp, while the longest consisted of 15,795 bp) and number of exons, which ranged from 2 to 18 exons. Most genes (32 out of 36) have both untranslated regions (UTRs). The *CsHDAC* genes also differed in length (the shortest transcript consisted of 2932 bp, while the longest consisted of 20,916 bp), but less significantly than *CsHATs*. The number of exons also varied from 3 to 18 exons. All genes have both UTRs.

### 3.4. Physicochemical Properties of CsHATs and CsHDACs

Physicochemical analysis and subcellular localization of the identified proteins were performed using the EMBOSS PEPSTATS and WoLF PSORT tools, respectively (Table 2). The proteins belonging to the CsHDAC family ranged in length from 296 aa to 659 aa and in molecular weight from 31.7 kD to 73.2 kD. The theoretical isoelectric point (pI) of the CsHDACs exhibited a range of values, from 4.4 to 7.5. Proteins of the CsHAT family showed a greater variety of parameters, with lengths from 113 aa to 1707 aa and molecular weights ranging from 12.5 kD to 192.6 kD. The theoretical isoelectric point (pI) of CsHATs ranged from 4.8 to 9.9. The complete results of the analysis are included in Supplementary Table S3. In terms of subcellular localization, the majority of CsHDACs were identified in the nucleus, as well as in the cytoplasm and chloroplast, whereas CsHATs were mainly located in the nucleus and cytoplasm.

### 3.5. Chromosomal Location of CsHDAC and CsHAT Genes

**Figure 5:**
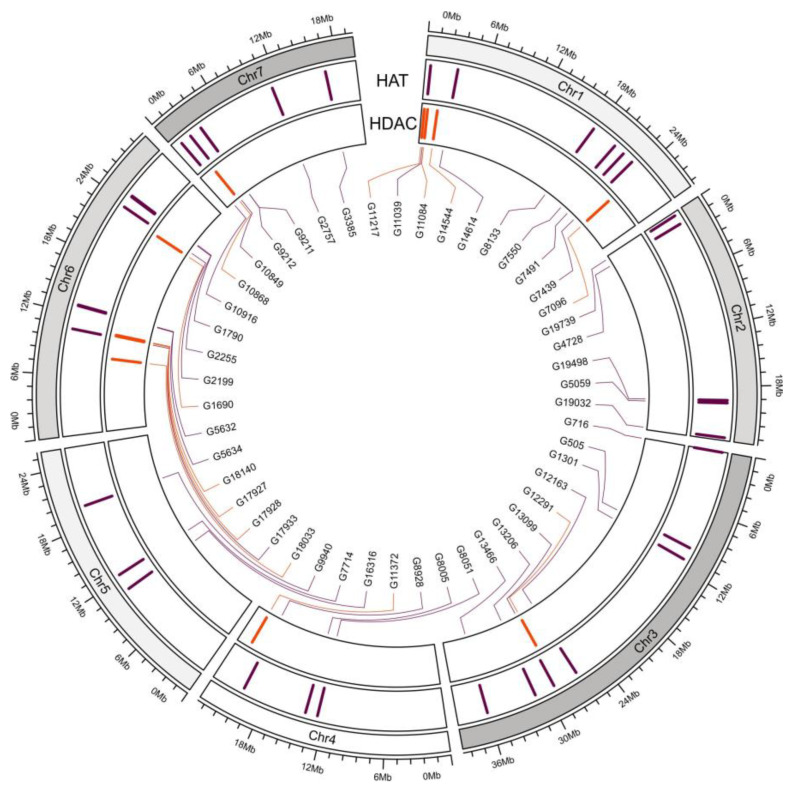
Chromosomal location of *CsHAT* and *CsHDAC* genes in cucumber B10v3. The position of the genes on each chromosome is indicated by bars—*CsHATs* in purple and *CsHDAC* in orange.

The genome annotation information (Table 2) was used to create a chromosome map of the *CsHDACs* and *CsHATs*, with all 48 members showing specific locations (Figure 5). The chromosomal distribution of 12 *CsHDACs* was found to be irregular, with the genes present on chromosomes 1, 3, 4, 6, and 7. The largest number—four CsHDAC genes—is present on chromosomes 1 and 6. Two CsHDACs are present on chromosome 4, while one gene each is present on chromosomes 3 and 7. The 36 CsHAT members were distributed on each chromosome. Seven genes are distributed on chromosome 3, six each on chromosomes 1 and 7, five *CsHAT* genes are present on chromosomes 2 and 5, and three genes each on chromosomes 4 and 5.

### 3.6. Expresional Analysis

**Figure 6:**
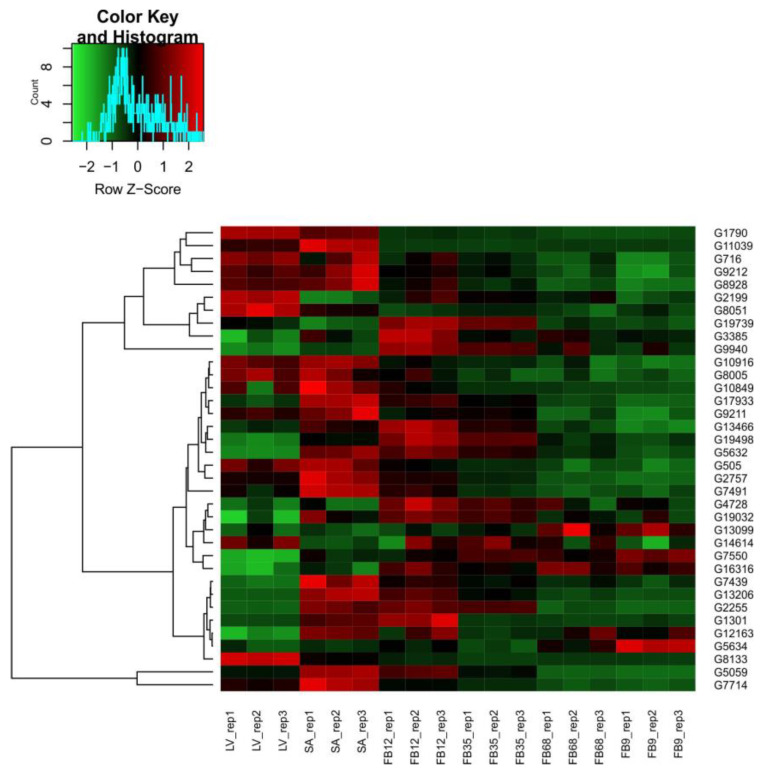
Heatmap illustrating CsHAT expression profiles across different samples: leaves (LV), shoot apexes (SA), and flower buds at different developmental stages—1–2 mm (FB12), 3–5 mm (FB35), 6–8 mm (FB68), and 9 mm (FB9). The color gradient ranges from red, indicating higher expression levels, to green, representing lower expression levels, with values in TPM.

**Figure 7:**
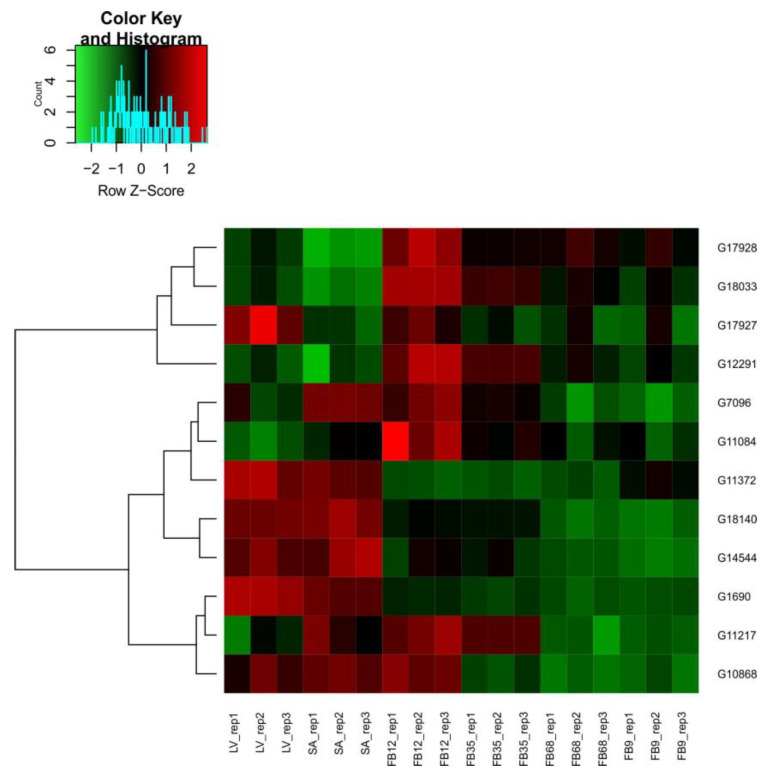
Heatmap illustrating *CsHDAC* expression profiles across different samples: leaves (LV), shoot apexes (SA), and flower buds at various developmental stages—1–2 mm (FB12), 3–5 mm (FB35), 6–8 mm (FB68), and 9 mm (FB9). The color gradient ranges from red, indicating higher expression levels, to green, representing lower expression levels, with values in TPM.

As a result of the analysis of the RNA-seq data for genes classified as acetyltransferases and deacetylases (Supplementary Table S4), heatmaps showing the expression profiles of these two groups during flower bud development were prepared. Samples from leaves, shoot apex, and flower buds at different developmental stages (1–2 mm, 3–5 mm, 6–8 mm, and 9 mm), in triplicate, were used in the analysis. Figure 6 presents the expression profile of *CsHATs*, while Figure 7 shows the expression profile of *CsHDACs*.

Visualizations of the expression profile revealed differences between the early stages of flower bud development (shoot apex and 1–2 mm) compared to the later stages (3–5 mm, 6–8 mm, 9 mm) for both acetyltransferases and deacetylases. The leaves represent a distinct group whose expression profile shows similarities to both the early and late stages of flower bud development.

**Figure 8:**
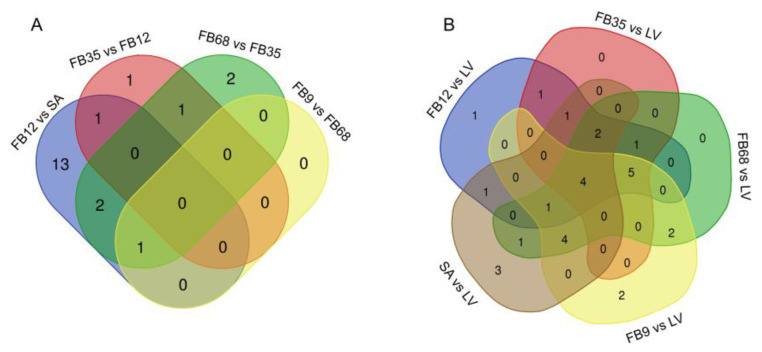
Venn diagram showing distribution of *CsHATs* and *CsHDACs* with differential expression exhibiting significant changes in comparisons between: (**A**) different developmental stages of flower buds: shoot apexes (SA) and flower buds of following lengths: 1–2 mm (FB12), 3–5 mm (FB35), 6–8 mm (FB68), and 9 mm (FB9) (**B**) developmental stages of flower buds (shoot apexes (SA) and flower buds of following lengths: 1–2 mm (FB12), 3–5 mm (FB35), 6–8 mm (FB68), and 9 mm(FB9)), and leaves (LV).

Among the genes classified as acetyltransferases and deacetylases, only a subset exhibited statistically significant changes in expression. Specifically, 26 acetyltransferase genes showed significant changes, compared to five genes among the deacetylases. Venn diagrams (Figure 8) illustrate the overlap of differentially expressed genes (DEGs) across various comparisons for both CsHATs and CsHADCs together. The corresponding gene identifiers are listed in Supplementary Table S5. Notably, 13 *CsHATs* showed unique changes in expression only in the comparison between the 1–2 mm flower buds and the shoot apexes (Figure 8A). The highest number of *CsHAT* or *CsHDAC* genes with differential expression during flower bud development was identified in the comparison between the 1–2 mm stages and the apexes, totaling 17 genes, of which 13 were unique to this comparison.

### 3.7. Promoter Analysis

**Figure 9:**
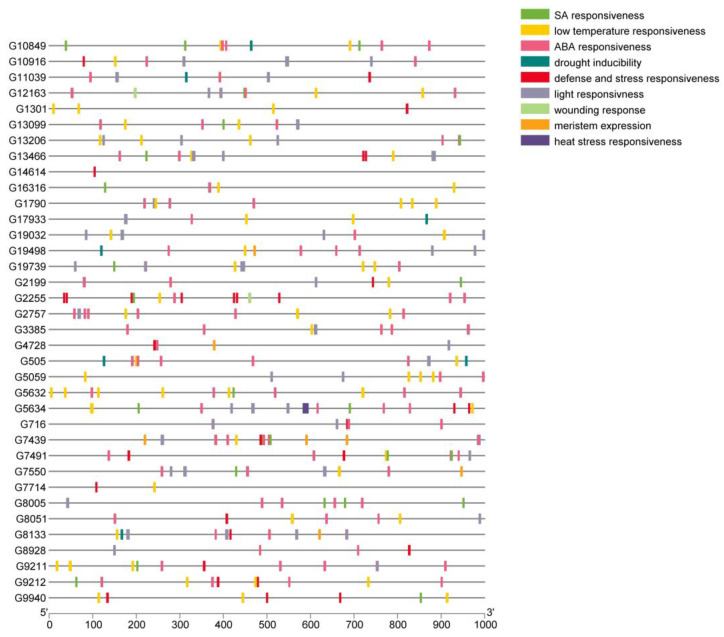
*Cis*-acting elements in the promoters of *CsHAT* genes. Distribution of *cis*-acting elements divided into major classes according to their function, in regions of 1000 bp upstream of the TSS. To improve clarity, the core elements have been omitted from the visualization.

The analysis of the promoter region of genes—identification of the *cis*-acting elements, their quantity and arrangement, may indicate the function and regulatory mechanisms of genes. The core promoter elements like TATA-box and CAAT-box, represented on average 65% of all elements found in *CsHAT* genes and 55% in *CsHDACs* genes (Figure 9). Besides core elements, we found *cis*-acting elements involved in the light response (for example ACE, Box4, GATA-motif, G-box), dehydration responsiveness (like MYB binding site or DRE), abscisic acid responsiveness (like ABRE, MYB), salicylic acid responsiveness (like as-1), low-temperature responsiveness (MYC), heat stress responsiveness (HSE1), defense and stress responsiveness (like W-box), wounding response (box S), and also elements involved in meristem expression (CAT-box) and differentiation of the palisade mesophyll cells (HD-Zip 1). The detailed results are presented in Supplementary Table S6.

**Figure 10:**
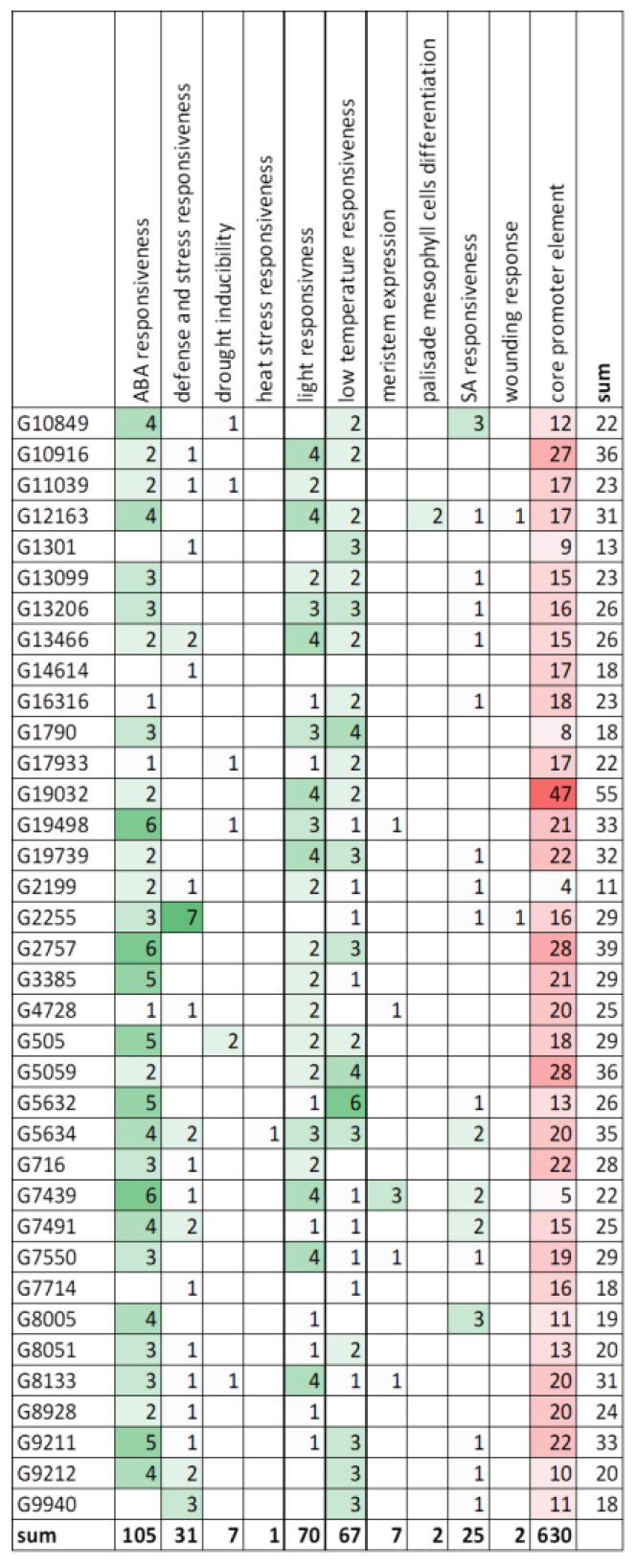
Number of *cis*-active elements in the promoters of *CsHAT* genes. The colors indicate the more abundant *cis*-active elements, green color indicate *cis*-elements, red color indicates core elements.

The analysis of the promoter regions of *CsHATs* revealed that the *cis*-acting elements involved in ABA responsiveness were the most abundant and accounted for 33.1% of all *cis*-elements found, excluding core promoter elements (Figure 10). The next most abundant groups were light responsiveness elements (22.1%) and low-temperature responsiveness elements (21.1%).

**Figure 11:**
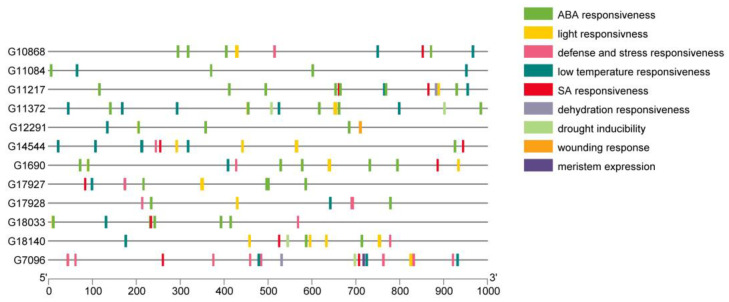
*Cis*-acting element analysis in the promoters of *CsHDAC* genes. Distribution of *cis*-acting elements divided into major classes according to their function, in regions of 1000 bp upstream of the TSS. To improve clarity, the core elements have been omitted from the visualization.

**Figure 12:**
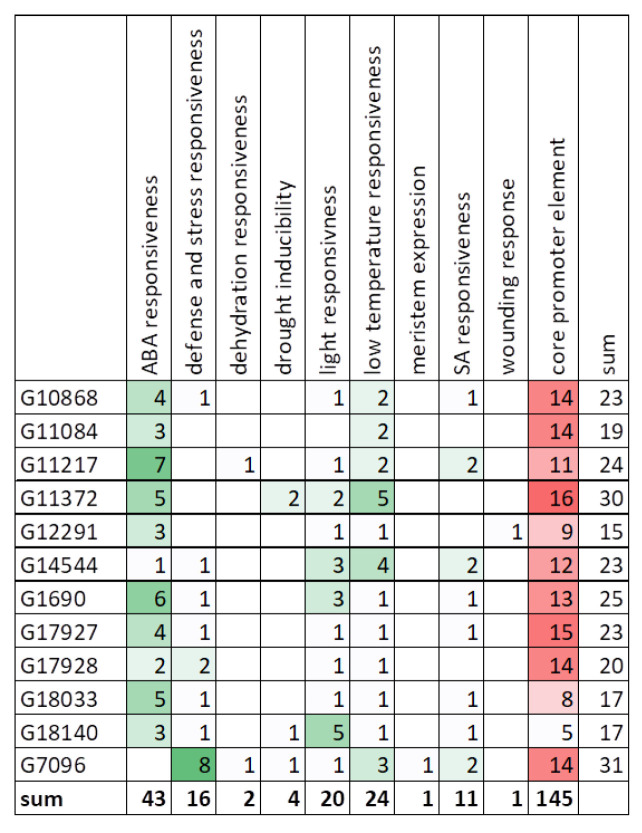
Number of *cis*-active elements in the promoters of *CsHDAC* genes. The colors indicate the more abundant *cis*-active elements, green color indicate *cis*-elements, red color indicates core elements.

The analysis of the promoter regions of *CsHDACs* showed that *cis*-acting elements involved in ABA responsiveness were also most abundant in these genes (Figure 11). They accounted for 35.2% of all *cis*-elements found (excluding core promoter elements). The next highly represented groups were elements involved in low-temperature responsiveness (19.7% of all elements) and light responsiveness elements (16.4%) (Figure 12).

## 4. Discussion

Histone modification plays a critical role in plant growth and development. Histone acetyltransferases and deacetylases contribute significantly to various plant physiological and metabolic processes, including chromatin remodeling and transcription regulation. These processes are essential not only for general growth but also for regulating genes involved in determining reproductive structures and processes [35]. Importantly, histone modifications are also implicated in plant sex determination. For example, reprogramming histone H3 lysine methylation has been shown to affect the expression of genes critical for sex-specific development during gametogenesis. Such modifications can influence the differentiation of male and female gametophytes, ultimately guiding sex determination and reproduction in plants [36,37].

In this study on *Cucumis sativus*, 12 HDAC and 36 HAT proteins were identified. Among these, the HDA family was the most prominent in the CsHDACs, while the HAG family predominated in the CsHATs. This distribution pattern is consistent with results from other plant species such as *Arabidopsis thaliana* [12], *Solanum lycopersicum* [20], *Triticum aestivum* [38], and *Vitis vinifera* [39]. Both CsHDACs and CsHATs contain family-specific structural domains [36,37]. In addition, the family members exhibit similar protein sequence lengths, motif compositions, and gene structures, suggesting a close phylogenetic relationship.

The expression profiles of acetyltransferase and deacetylase genes during flower bud development, visualized through RNA-seq heatmaps, provide insights into the regulatory dynamics of these two groups of genes. The findings reveal distinct stage-specific gene expression patterns, emphasizing the dynamic role of histone-modifying enzymes in floral development. Acetyltransferase genes showed significant shifts in expression between early and late developmental stages. Their activity was distinct during the shoot apex and 1–2 mm flower bud stages, suggesting a potential role in initiating developmental programs. In contrast, later stages (3–5 mm, 6–8 mm, and 9 mm flower buds) displayed altered acetyltransferase expression, possibly supporting differentiation and maturation processes. Similarly, deacetylase genes showed pronounced differences between the early and late stages of flower bud development, reflecting their involvement in repressing or fine-tuning gene expression as the flower bud progresses through development. It is worth mentioning that previous studies on cucumber flower bud development indicate that at early stages of development when flower buds are about 1 mm long, the initiation of particular primordia growth and flower sex differentiation begins. In the later stages, the growth of primordia is continued [40].

Interestingly, leaf samples displayed an intermediate pattern of expression, sharing similarities with both the early and late stages of flower bud development. This suggests overlapping regulatory mechanisms or retention of developmental cues common to vegetative and floral tissues. The different expression profiles of acetyltransferases and deacetylases during flower bud development are consistent with the literature findings that highlight their key roles in regulating developmental processes. For instance, studies have demonstrated that histone acetyltransferases are crucial for chromatin remodeling and gene expression during plant growth. In *Vitis vinifera*, HATs have been shown to regulate the expression of genes associated with developmental transitions, indicating their role in organ differentiation [39]. Studies on *HATs* in *Triticum aestivum* also indicate the role of these genes in plant development [1].

Similarly, histone deacetylases have been implicated in stage-specific expression during plant development. Research on rice HDAC families revealed distinct expression patterns, correlating with specific developmental stages and stress responses. This emphasizes the dual role of HDAC in controlling gene silencing and developmental fine-tuning [41].

These examples provide further evidence of the functional importance of dynamic acetylation and deacetylation processes, supporting the observed stage-specific expression profiles in flower bud development. Such regulation ensures proper progression through developmental stages, balancing activation and repression of critical genetic pathways [14,42].

Recent studies have revealed the involvement of HDAC proteins: HDA6 and HDA5 in the regulation of flowering by the autonomic pathway [43]. In particular, *hda6* mutants exhibit delayed flowering phenotypes in both long-day and short-day conditions, with their flowering time being dependent on the presence of FLC [44]. 

In bananas, HDA1 has been shown to interact with ERF11, a component of the ethylene signaling pathway, to delay fruit ripening. This delay also occurs through repression of the ethylene biosynthesis gene *MaACO1* and the expansin genes [45]. Another histone deacetylase, MaHDA6, plays a role in promoting ethylene signaling and fruit ripening by inhibiting ERF11/15 genes [46]. Similarly, in tomatoes, different HDACs have different effects on fruit ripening. SlHDA3 acts as a ripening inhibitor by regulating ethylene biosynthesis [47], whereas SlHDT3 stimulates ethylene and carotenoid accumulation, contributing to fruit ripening [48]. In papayas, CpHDA3, an RPD3-type histone deacetylase, forms a repressor complex with ERF9 [49]. The involvement of HDACs in ethylene biosynthesis and signaling pathways is also important in cucumber floral sex determination, in which ethylene plays a key role as a major hormone [50].

It can be seen that in the 1000 bp of the analyzed promoter region, the most abundant motifs are the motifs common to CsHAT/CsHDAC, which are 105/43 (32/11 genes), 70/20 (29/11 genes), and 67/24 (30/12 genes), respectively for the processes of ABA responsiveness, light responsiveness, and low-temperature responsiveness. The above three processes are mainly related to cell physiology and concern many aspects of ABA use, including sex development [51,52].

These results highlight the complex interaction between acetylation and deacetylation during developmental transitions, which may have a real connection with ethylene signaling during cucumber flower morphogenesis. This provides a foundation for further research into their specific functional roles in floral organogenesis.

## 5. Conclusions

The role of histone modifications, particularly acetylation and deacetylation, is fundamental for plant growth, development, and reproduction. Histone acetyltransferases (HATs) and deacetylases (HDACs) coordinate critical regulatory processes through chromatin remodeling and transcriptional control. The stage-specific expression of *CsHAT* and *CsHDAC* genes during flower bud development underscores their dynamic involvement in initiating, fine-tuning, and completing developmental transitions. In *Cucumis sativus*, the identification of diverse HDAC and HAT families highlights conserved patterns and unique subfamily features that are consistent with other plant species. By modulating histone H3 lysine methylation and other epigenetic marks, they influence gametophyte differentiation and gene expression critical for male and female organogenesis. The results of RNAseq analysis showed that HATs and HDACs play an important role in flower bud development and growth. Overall, the interplay between histone acetylation and deacetylation ensures a balanced progression of gene expression programs, enabling plants to adapt their developmental pathways to internal and external signals. These findings provide a basis for deeper investigations into epigenetic mechanisms regulating floral organogenesis and sex determination, with potential implications for crop improvement and yield optimization.

## Figures and Tables

**Table 1:** Summary of CsHAT and CsHDAC proteins identified in B10v3 cucumber genome, compared to available data for *Arabidopsis thaliana* and *Solanum lycopersicum*.

Type	Family	Species		
		*Cucumis sativus* *B10v3*	*Arabidopsis thaliana*	*Solanum lycopersicum*
HAT	HAG	28	3	23
	HAM	1	2	1
	HAC	7	5	4
	HAF	0	2	1
	Total	36	12	29
HDAC	HDA	9	9	7
	HDT	3	4	3
	SRT	0	2	2
	Total	12	15	12

**Table 2:** List of *CsHATs* and *CsHDACs* genes identified in B10v3 genome and the physicochemical properties of the encoded proteins.

Family	Gene ID	Chr. No	Chr Location Start/Stop	String	Length Genomic	Length Peptide	Molecular Weight (kD)	Isoelectric Point (Mw)	Localization
Histone acetyltransferases (HATs)									
HAG family									
HAG	Cucsat.G7439	Chr1	ctg1528:2195363-2199742	+	4380	374	43.3	7.5	nuclear
HAG	Cucsat.G7491	Chr1	ctg1528:3357562-3360155	+	2594	299	34.6	9.3	cytoplasmic
HAG	Cucsat.G7550	Chr1	ctg1528:4490591-4496659	+	6069	516	58.5	5.9	nuclear
HAG	Cucsat.G8133	Chr1	ctg1557:892275-894304	+	2030	397	45.3	8.3	cytoplasmic
HAG	Cucsat.G11039	Chr1	ctg1740:323653-327391	+	3739	564	63.6	8.5	cytoplasmic
HAG	Cucsat.G14614	Chr1	ctg1869:2371776-2380341	+	8566	269	30.3	9.4	nuclear
HAG	Cucsat.G4728	Chr2	ctg1227:2564029-2569251	+	5223	164	18.6	9.0	cytoplasmic
HAG	Cucsat.G19032	Chr2	ctg35:283689-291641	−	7953	255	29.1	8.7	cytoplasmic
HAG	Cucsat.G19739	Chr2	ctg4:329471-333655	+	4185	464	52.1	4.8	cytoplasmic
HAG	Cucsat.G1301	Chr3	ctg1:11167598-11170238	+	2641	415	47.3	8.4	cytoskeleton
HAG	Cucsat.G505	Chr3	ctg1:9926620-9928112	−	1493	169	19.3	6.0	cytoplasmic
HAG	Cucsat.G12163	Chr3	ctg1837:1420082-1422790	−	2709	409	46.4	8.8	cytoplasmic
HAG	Cucsat.G13099	Chr3	ctg1838:168257-179247	+	10,991	163	18.5	5.7	nuclear
HAG	Cucsat.G8005	Chr4	ctg1556:2247813-2251525	−	3713	113	12.5	9.0	nuclear
HAG	Cucsat.G8051	Chr4	ctg1556:3460680-3465076	−	4397	248	27.8	6.1	chloroplast
HAG	Cucsat.G8928	Chr4	ctg1635:1972081-1987877	−	15,797	1403	155.3	6.1	nuclear
HAG	Cucsat.G9940	Chr5	ctg1673:1987918-1994420	+	6503	299	34.2	8.3	chloroplast
HAG	Cucsat.G16316	Chr5	ctg2246:313272-316828	−	3557	196	22.4	6.2	cytoplasmic
HAG	Cucsat.G1790	Chr6	ctg1002:2300165-2307083	−	6919	634	70.1	8.3	cytoplasmic
HAG	Cucsat.G2199	Chr6	ctg1002:985444-1000064	+	14,621	1030	116.1	8.0	cytoplasmic
HAG	Cucsat.G2255	Chr6	ctg1002:2192294-2194872	+	2579	410	46.2	8.9	cytoplasmic
HAG	Cucsat.G5632	Chr6	ctg1299:318309-325444	+	7136	1432	161.5	8.1	nuclear
HAG	Cucsat.G5634	Chr6	ctg1299:396719-398897	+	2179	415	47.8	8.7	nuclear, cytoplasmic
HAG	Cucsat.G17933	Chr6	ctg3345:1507356-1519041	+	11,686	972	107.2	6.2	nuclear
HAG	Cucsat.G2757	Chr7	ctg1041:822751-825505	−	2755	271	30.7	8.1	chloroplast
HAG	Cucsat.G3385	Chr7	ctg1041:6256432-6258342	+	1911	220	24.8	7.9	extracellular
HAG	Cucsat.G10849	Chr7	ctg1681:902951-910263	−	7313	225	25.9	5.7	cytoplasmic
HAG	Cucsat.G10916	Chr7	ctg1681:2018378-2026590	−	8213	297	33.1	8.8	chloroplast
HAC family									
HAC	Cucsat.G5059	Chr2	ctg1227:2823211-2832569	−	9359	395	44.2	9.5	nuclear
HAC	Cucsat.G19498	Chr2	ctg4:1282651-1287430	−	4780	423	47.8	7.0	nuclear
HAC	Cucsat.G716	Chr3	ctg1:42742-56672	+	13,931	1707	192.6	8.1	nuclear
HAC	Cucsat.G13206	Chr3	ctg1838:2203445-2207740	+	4296	721	80.5	9.5	nuclear
HAC	Cucsat.G13466	Chr3	ctg1838:7194802-7198169	+	3368	355	40.4	8.6	cytoplasmic
HAC	Cucsat.G9211	Chr7	ctg1658:1590288-1598827	+	8540	127	14.4	4.8	nuclear
HAC	Cucsat.G9212	Chr7	ctg1658:1599861-1614805	+	14945	1365	149.8	9.9	nuclear
HAM family									
HAM	Cucsat.G7714	Chr5	ctg1546:298050-304738	+	6689	495	57.3	8.6	chloroplast
Histone deacetylases (HDACs)									
HDA family									
HDA	Cucsat.G11084	Chr1	ctg1740:159401-162484	−	3084	465	52.3	5.8	cytoplasmic
HDA	Cucsat.G11217	Chr1	ctg1780:533431-536277	+	2847	375	41.0	5.8	cytoplasmic, cytoskeleton
HDA	Cucsat.G7096	Chr1	ctg1528:914219-935116	−	20,898	605	66.4	6.8	nuclear
HDA	Cucsat.G14544	Chr1	ctg1869:945967-954231	+	8265	659	73.2	5.3	chloroplast
HDA	Cucsat.G1690	Chr4	ctg1002:373456-379733	−	6278	442	48.2	6.6	chloroplast
HDA	Cucsat.G11372	Chr4	ctg1798:64712-72066	−	7355	352	38.9	7.5	cytoskeleton
HDA	Cucsat.G18033	Chr6	ctg3345:3960732-3965777	+	5046	492	55.6	5.0	nuclear
HDA	Cucsat.G18140	Chr6	ctg3345:1224920-1231792	−	6873	440	50.4	5.1	mitochondrial
HDA	Cucsat.G10868	Chr7	ctg1681:1172904-1178169	−	5266	471	53.3	5.7	cytoplasmic
HDT family									
HDT	Cucsat.G12291	Chr3	ctg1837:3557001-3565310	−	8310	318	34.6	4.4	nuclear
HDT	Cucsat.G17927	Chr6	ctg3345:1319359-1323178	+	3820	296	31.7	4.4	nuclear
HDT	Cucsat.G17928	Chr6	ctg3345:1323822-1327471	+	3650	571	61.6	4.8	nuclear

## Data Availability

Publicly available datasets were analyzed in this study and are referenced in the cited articles.

## Supplementary Materials

The following supporting information can be downloaded at: https://www.mdpi.com/article/10.3390/genes16020127/s1, Table S1: Results of the InterProScan search, Figure S1, Results of phylogenetic analysis for HAT and HDAC domains, Table S2: Gene structure analysis, Table S3: Results of EMBOSS PEPSTATS analysis, Table S4: Results of expressional analysis, Table S5: Venn diagram results tables, Table S6: Results of the PlantCARE promoter analysis.
