# Supplementary material for: Genome-Wide Identification and Characterization of Histone Acetyltransferases and Deacetylases in Cucumber, and Their Implication in Developmental Processes
Source: Genes (Basel). 2025 Jan 23;16(2):127. doi: 10.3390/genes16020127 (PMC11855351; doi:10.3390/genes16020127)
Supplement: Supplementary file 1 [file genes-16-00127-s001.zip › Figure S1.pdf]

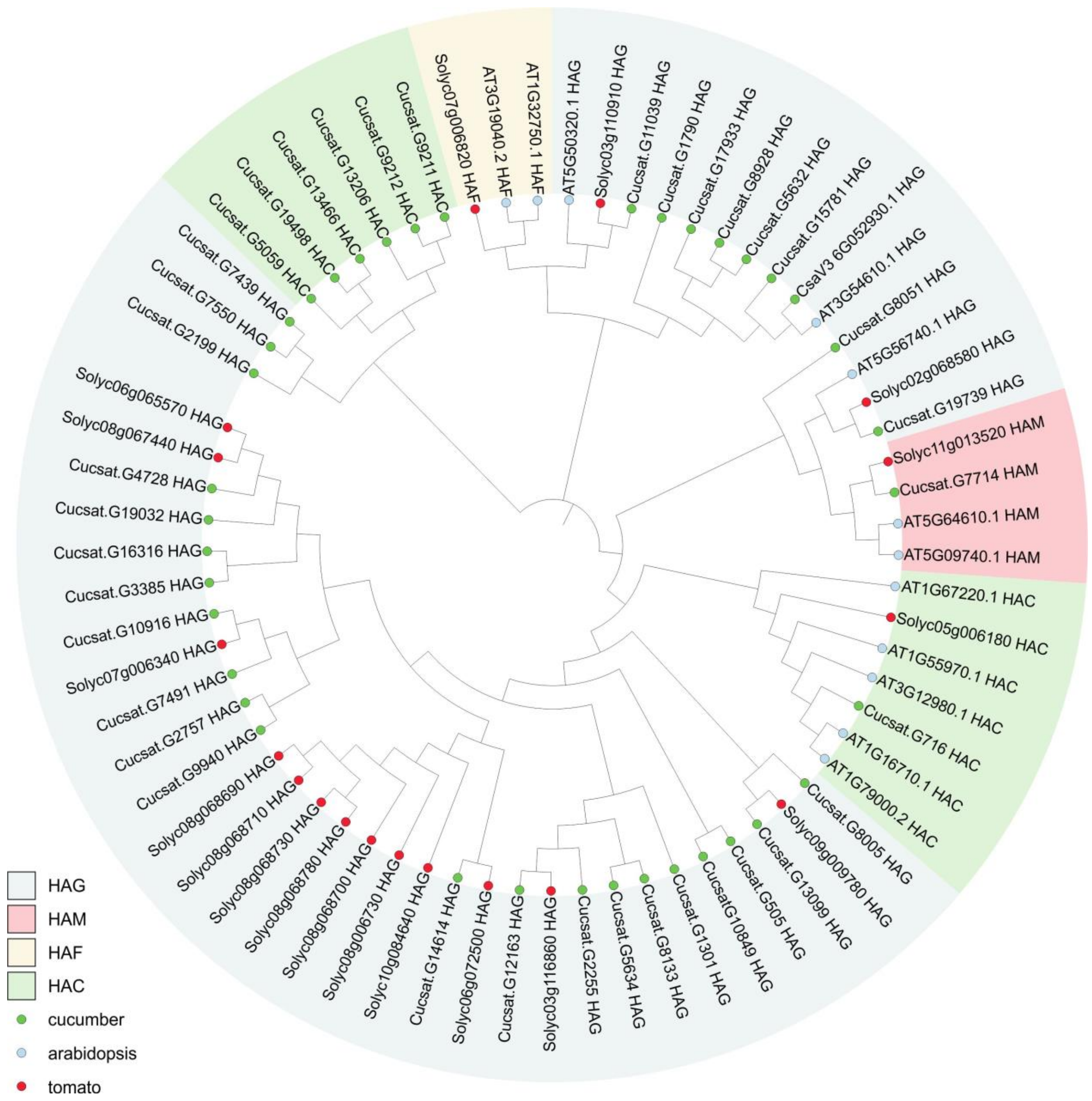

Figure S1A. Phylogenetic analysis of HAT proteins from Cucumis sativus (36 proteins, marked with green circles), Arabidopsis thaliana (12 proteins, marked with blue circles) and Solanum lycopersicon (29 proteins, marked with red circles). The phylogenetic tree was built with the neighbour-joining method using only sequences of specific domains. The subfamilies of HAG, HAM, HAF and HAC were marked with different colors.

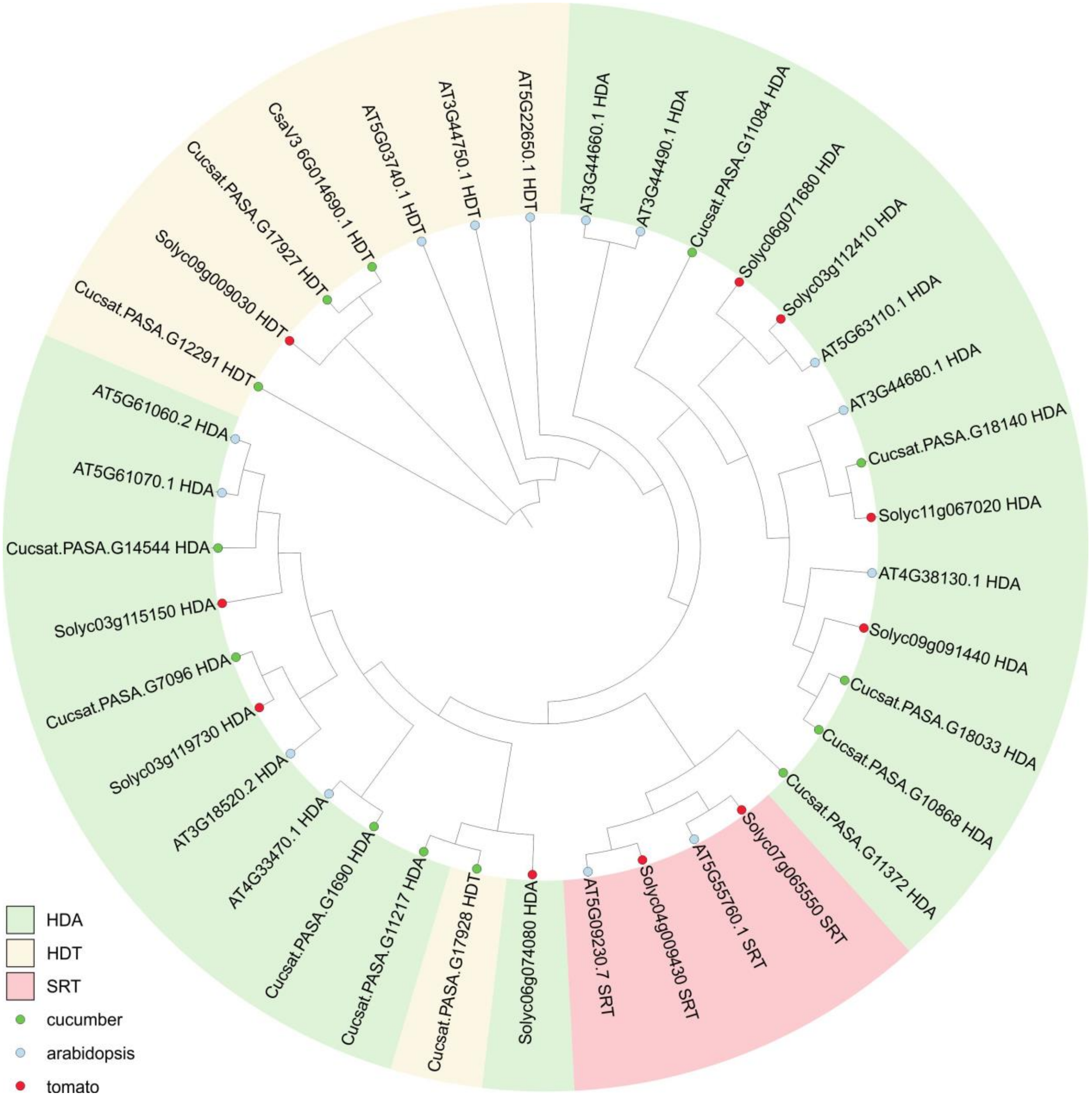

Figure S1B. Phylogenetic analysis of HDAC proteins from Cucumis sativus (12 proteins, marked with green circles), Arabidopsis thaliana (15 proteins, marked with blue circles) and Solanum lycopersicon (12 proteins, marked with red circles). The phylogenetic tree was buil with the neighbour-joining method method using only sequences of specific domains. The subfamilies of HDA, HDT and SRT were marked with different colors.
